# Red Blood Cell Distribution Width, Erythrocyte Indices, and Elongation Index at Baseline in a Group of Trained Subjects

**DOI:** 10.3390/jcm13010151

**Published:** 2023-12-27

**Authors:** Gregorio Caimi, Melania Carlisi, Rosalia Lo Presti

**Affiliations:** 1 Department of Health Promotion and Child Care, Internal Medicine and Medical Specialties, University of Palermo, 90127 Palermo, Italy; melaniacarlisi@yahoo.it; 2 Department of Psychology, Educational Science and Human Movement, University of Palermo, 90127 Palermo, Italy; rosalia.lopresti@unipa.it

**Keywords:** regular exercise, red blood cell distribution width, erythrocyte indices, erythrocyte deformability, trained subjects

## Abstract

Background: Regular exercise elicits adaptive changes in several organs and physiological processes, including erythrocyte properties. Methods: In a group of 79 subjects (62 men and 17 women; mean age 31.37 ± 10.19 years) who trained several times a week as they practiced amateur sports, we evaluated the elongation index, markers of erythrocyte deformability, red blood cell distribution width (RDW), indicators of erythrocyte anisocytosis, hematocrit, hemoglobin, and the main erythrocyte indices (MCV, MCH, MCHC) in basal conditions. Results: In comparison with a group of healthy, but not training, volunteers, the values of the elongation index, and not the RDW, are increased, and this datum is accompanied by an increase in MCV and MCHC, likely related to an increased presence of circulating young erythrocytes in training subjects. We also divided the same group according to the median of the VO_2_max, observing that the subgroup above the median shows both an increase in the elongation index values and a decrease in MCH and MCHC. Conclusions: In trained subjects, there is no correlation between the values of the elongation index and the RDW, while the interrelations among the elongation index, RDW, and main erythrocyte indices appear to be of particular interest and of a certain complexity.

## 1. Introduction

For a long time now, the evaluation of the peripheral blood count has been entrusted to automated blood counters, which reach a higher level of precision than manual counting. Among the blood count parameters, the red cell distribution width (RDW) is of particular significance, favoring a complete evaluation of the erythrocyte component. The RDW is a dimensionless quantitative value that reflects the variation in cell size in the erythrocyte population. In different clinical conditions, it is possible to observe an increase in the RDW value, and the main causes could be a decrease in red blood cell (RBC) mean volume, increased reticulocyte volume variance, increased heterogeneity in the RBC volume in the peripheral circulation, and delayed erythrocyte clearance [1,2]. Furthermore, any change in RDW should be assessed, taking into account the hemoglobin values, erythrocyte number, and mean corpuscular volume, respectively.

The erythrocyte deformability is a hemorheological determinant that plays an undisputed role in the microcirculation, where high shear rates are in force. It depends on surface/volume ratio of the erythrocyte, internal viscosity (mainly related to the concentration of cytosolic calcium, erythrocyte organic phosphates (ATP and 2.3 DPG) and changes in hemoglobin), and on the dynamic membrane properties [3].

The deformability, in addition to allowing the erythrocyte to easily cross microvascular districts of a few microns, plays a key role in the supply of oxygen to tissues.

In subjects who practice sporting activities both at the amateur and elite level, as well as in those who carry out physical activity in a constant and particularly intense way, it is likely that, in basal conditions, the RDW is not increased, unless they are anemic; this is because one of the doctrinal assumptions of the RDW increasing, in the absence of hematological diseases, is the increased survival of red blood cells. The negative correlation between the RDW and muscle-strengthening activities [4] and between physical activity and the RDW is already known [5], although few and often fragmentary literature data on this topic are available. Several authors have, in fact, concluded that physical activity tends to reduce this erythrocyte index [6,7]. The behavior of this index is different at the end of an acute and extremely intense exercise session, because in the hours following the event, a reduction in hemoglobin levels can be observed [8,9]. In a dated analysis [10] carried out on 2143 children and adolescents aged between 12 and 20 years and performed between 2003 and 2006 under the NHANES project, sedentary and physical activity levels were assessed in relation to RDW behavior, and it was found that in boys, and not in girls, the sedentary activity was positively associated with the RDW, while physical activity was negatively associated with the erythrocyte index.

Over the years, it has been shown that exercise affects the hemorheological profile in a completely different way depending on whether it is practiced regularly or is carried out over a short period and with high intensity. What has been said, in agreement with other authors [11], underlines how exercise, in relation to the hemorheological pattern, configures very complex hemodynamic, metabolic, and hemorheological frameworks.

So far, the effects of exercise on erythrocyte deformability do not seem univocal: sometimes high-intensity exercises determine its reduction, while, in other cases, concerning training subjects, an increase is evident; finally, in some studies, there are, instead, no appreciable variations in this hemorheological determinant. During high-intensity exercises, the decrease in erythrocyte deformability can be attributed to changes in hydrogenionic concentration, due to the increase in lactic acid, changes in plasma osmolarity, or the unbalanced increase in reactive oxygen species. In different groups of subjects practicing different sports, several authors have observed, under basal conditions, an increase in red blood cell deformability [12,13,14,15,16,17,18,19,20,21,22,23] although others have described a reduction in this hemorheological determinant [24,25,26], and others have not observed any variation in this parameter [27,28,29]. In athletes, an accelerated turnover of erythrocytes is found, and this leads to a very high percentage of younger cellular elements that perhaps explains the increase in erythrocyte deformability in athletes [30,31].

From the point of view of clinical hemorheology, it leads us to seriously reflect on how important it is not to exclude that, in the determinism of the deformability of the red blood cells, we must take into due consideration the degree of anisocytosis that is expressed by this erythrocyte index. Until now, the data obtained in relation to the study of correlation between these two parameters have not converged in the literature. The first response concerned a cohort of 293 adults with an average age of 71.1 ± 13.3 participating in a longitudinal aging study, in which a significant negative correlation was found between the RDW and the elongation index evaluated at the shear stress of 3 Pa [32]. This correlation became significantly more marked when the entire cohort excluded the group of anemic subjects.

Considering the above, in this paper we want to examine the behavior of RDW and erythrocyte deformability in a group of regularly training subjects, who are non-professionals performing regular sports activities such as endurance, power and mixed activities. In particular, we evaluate whether there is a direct relationship between them and how the exercise acts on some erythrocyte (MCV, MCH, MCHC) and non-erythrocyte parameters.

## 2. Materials and Methods

### 2.1. Population

We enrolled 79 subjects (62 men and 17 women; mean age 31.37 ± 10.19 years; range 18–57 years), non-professional athletes, who practiced regular physical sports training, three or four times a week. Thirty of these subjects practiced endurance sports, twenty-five of these subjects performed mixed sports, and twenty-four of them practiced power sports. In this group of non-professional athletes, the VO2max was 33.14 ± 10.86 mL/Kg/min. The control group is represented by 25 subjects (19 men and 6 women with a mean age of 33.04 ± 5.77 years; range 24.00–48 years), who did not engage in any physical activity, In this control group, the VO2max was 22.11 ± 5.77 mL/Kg/min. As demonstrated by clinical history, objective examination, electrocardiography, and routine hematological and urine analyses, all subjects did not have cardiovascular or other medical diseases. No subjects had taken medication in the previous weeks, and no subject had been subjected to dietary restrictions prior to the study. VO2max was evaluated by a computerized breath-by-breath analyzing system (oxycon Delta; Viasys Jaeger, Hoechberg, Germany). Informed consent was obtained from each participant.

### 2.2. Methods

In fasting venous blood, we evaluated hematocrit (Ht), obtained using an automated hematology analyzer, hemoglobin, expressed in gr/L and evaluated with the automated blood count, mean corpuscular volume (MCV), expressed in fl, mean concentration hemoglobin (MCH), expressed in pg, mean corpuscular hemoglobin concentration (MCHC), expressed in gr/dL, RDW expressed as a percentage, and erythrocyte deformability, expressed as elongation index. To evaluate this latter hemorheological parameter, 30 µL of anticoagulated blood was mixed with 2 mL of dextran solution at a viscosity of 24 mPa. The measurement was performed using the Rheodyn SSD diffractometer of Myrenne, which measures the diffraction pattern of a laser beam passing through erythrocytes suspended in a viscous medium and deformed by a force with defined shear stress. We employed shear stress of 6, 12, 30, and 60 Pa. The erythrocyte deformation was expressed as elongation index [(EI) = (l − w/l + w) × 100], where l = length and w = width of the erythrocytes. All the parameters were evaluated in a time range between 3 and 5 days from the last training session.

### 2.3. Statistical Analysis

Statistical analysis was performed with the GraphPad Prism vers. 9.5 program. The data were presented as means and standard deviation. We have used the Student’s *t*-test for unpaired data to perform the comparison between the means. We have employed the Pearson’s test for the analysis of the different correlations. The null hypothesis was considered for values of *p* ≤ 0.05.

## 3. Results

First, we tabulated the means, standard deviation, and the ranges of all the considered parameters regarding this group of regularly training subjects who carry out non-professional sports activities. In this study, we considered, under basal conditions, the following parameters: hematocrit, hemoglobin level, MCV, MCHC, red blood cell distribution width (RDW%), and the values of the elongation index at the shear stress of 6, 12, 30, and 60 Pa, respectively (Table 1). Immediately after, we compared the mean values between the control group and the trained subjects, observing in the entire group of trained subjects a significant increase in the MCV and the MCH, associated with the elongation index. This comparison did not reveal any change in hematocrit, hemoglobin level, MCHC, and RDW (Table 2). We then divided the whole group of trained subjects according to the median of VO2max (30.00 (11.40) mL/kg/min), observing that in the subgroup that exceeds the median, there was an evident reduction of the MCH and the MCHC, associated with a significant increase in the elongation index. VO2max represents the maximum volume of oxygen consumed per minute (in milliliters) per kilogram of weight and it defines the personal cardiorespiratory and aerobic level. In our analysis, VO2max was calculated with a cardiopulmonary test, using a cycloergometer with load incremental steps until maximal aerobic capacity (VO2max) and assessed by a computerized breath-by-breath analyzing system (Oxycon Delta; Viasys Jaeger, Hoechberg, Germany). VO2max corresponded to the plateau in oxygen consumption, despite the increment in the workload.

**Figure 1:**
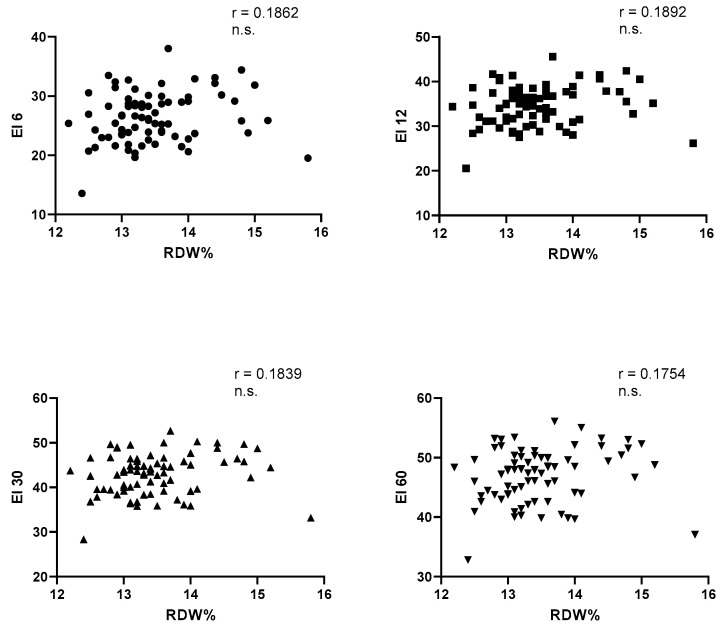
Correlations (Pearson’s test) between RDW% and EI at the shear stresses of 6, 12, 30, 60 Pa. RDW = red blood cell distribution width; EI 6 = elongation index at the shear stress of 6 Pascal; EI 12 = elongation index at the shear stress of 12 Pascal; EI 30 = elongation index at the shear stress of 30 Pascal; EI 60 = elongation index at the shear stress of 60 Pascal. Circle: EI 6 Pa; Square:EI 12 Pa; Triangle with lower base: EI 30 Pa; Triangle with upper base: EI 60 Pa.

On the other hand, no variation has been observed for all the other considered parameters (Table 3). In this group of trained subjects, no correlation was observed with each value of elongation index, respectively examined at shear stress of 6, 12, 30, and 60 Pa, and RDW (Figure 1). At this point, to clarify the parameters able to influence the RDW and the elongation index, we examined, with the Pearson test, the different statistical correlations (Table 4). Initially, we observed that RDW is negatively related to MCH and MCHC only. No correlation is recorded with the age of subjects, VO2max, hematocrit, the hemoglobin level, and with MCV. The behavior of the elongation index is quite different. It is negatively related to hematocrit, hemoglobin, and MCHC and positively related to MCV. No correlation exists between MCH and the elongation index values and between MCH and the age of the subjects. Of particular interest, but difficult to interpret, is the positive correlation between the values of VO2max and the elongation index evaluated at shear stress of 6 and 12 Pa.

## 4. Discussion

From the obtained data, the first consideration should concern the different trends of RDW and elongation index in the control group (sedentary subjects not trained) and the group of non-professional sports players, who perform a regular physical activity several times a week. The RDW does not distinguish the two groups, because it is a marker of erythrocyte anisocytosis, and it tends to increase, in the absence of an anemic condition, with the average life of the erythrocyte. Our observations agree with previous reported data [4,5,6,7,10]. The trend of RDW several hours after performing moderate aerobic exercise [8], or after performing an extreme run [9], is different. The behaviors of the elongation index with the four different shear stresses are quite different, which are increased in the group of trained subjects, confirming our previous data [19] and those from several other authors [12,13,14,15,16,17,18,20,21,22,23]. The increase in erythrocyte deformability in trained and amateur sports subjects plays a role in the transport of oxygen to tissues, especially in vascular districts where the highest shear rates are physiologically in force [33].

Another result of particular interest is the trend of some erythrocyte parameters such as MCV and MCH. Both these parameters are significantly increased in the trained subjects compared to the control group, and this feedback, not at all occasional, is based on what is now ascertained about the effects of exercise on erythrocyte production [34], on the regulation of the RBC volume [35,36], and on the whole erythropoiesis [37]. In fact, exercise, promoting bone marrow hyperplasia [38], can reduce the circulating level of proinflammatory cytokines [39,40], and it can vary the affinity and concentration of erythropoietin receptors [41], although the increase in concentration does not seem to be certain [42]. The changes in the hormonal profile (testosterone, growth hormone, and especially the insulin-like growth factor), observed in relation to exercise, [43,44] may stimulate erythrocyte production. Therefore, there are likely to be more and more young erythrocytes in circulation that simultaneously explain both the increase in erythrocyte deformability and the increase in MCV and MCH.

Of particular interest is what happens by dividing the group of trained subjects in relation to the median values of the VO2max. In this case, what is evident from the values of the elongation index is of pathophysiological interest. In fact, in the subgroup that exceeds the median, it is evident, even if at low statistical significance, an increase in the red blood cell deformability associated with a reduction in MCH and MCHC. It is possible to hypothesize that the decrease in these two erythrocyte parameters may be because, in the subgroup that exceeds the VO2max median, there is a clear tendency to reduce the value of total hemoglobin that helps to better explain the trend of both MCH and MCHC.

In this group of trained subjects, as could be expected, given the trend of the RDW and the values of the elongation index, there is no significant statistical correlation between them. A negative correlation could theoretically be expected since there are certainly young erythrocytes and, therefore, it was conceivable that the reduction of the RDW would be associated with higher elongation index values, confirming a higher erythrocyte deformability. So far, in addition to what Patel reported in 2013 [32], that described a negative correlation between RDW and the value of elongation index at shear stress of 3 Pa, other authors, in different clinical conditions, have found discordant data. In fact, some authors showed a positive correlation between these two parameters in hypertensive patients [45], in patients with acute myocardial infarction [46], and in subjects with metabolic diseases. Like what Patel [32] found, a negative correlation between RDW and elongation index occurred in subjects with thalassemic traits and in patients with hematological neoplasms [47].s

Is not always easy to interpret the results about the evaluated correlations in the group of trained subjects. According to others [48], the RDW is not related to the age of the subjects nor to the values of VO2max. However, in a large series of 1111 healthy subjects, other authors have observed a positive correlation between RDW and age [49]. RDW is also unrelated to hematocrit, hemoglobin, and MCV. Others, instead, in healthy subjects and in hypertensive adolescents [50], showed a negative correlation between RDW, hemoglobin, and MCV. RDW, in our group of trained subjects, is negatively related to MCH and MCHC; this last result is in line with what is described by Sun and co-workers and Vayá et al.

Much more articulated are the correlations between the values of the elongation index. According to the available literature data [51], the values of elongation index correlate negatively with hematocrit, hemoglobin, and MCHC and positively with MCV. This last datum appears to be a confirmation of the fact that, in trained subjects, the presence of young erythrocytes in a higher percentage than the norm is more than plausible. Of sure interest is what has been observed correlating the elongation index values with the VO2max. This correlation tends to show that the increase in VO2max corresponds to the increase in erythrocyte deformability and seems to converge with what other authors have found [52,53]. In our case studies, for some shear stresses (6 Pa and 12 Pa), this relationship between elongation index and VO2max is present, but there is also a negative correlation between MCH and VO2max and between MCHC and VO2max (data not shown).

## 5. Conclusions

In conclusion, in regularly trained subjects there is a significant increase in erythrocyte deformability associated with an increase in some erythrocyte parameters such as MCV and MCHC, expressions of an increment of young red blood cells that regular exercise entails. In the subdivision performed according to the values of the VO_2_max median, it is evident that in the subgroup above the median, there is an increase in red cell deformability associated with the decrease in MCH and MCHC. There is no statistical relationship between elongation index values and RDW. The findings that emerge from the study of the interrelationships between RDW, elongation index, and erythrocyte indices are not conclusive.

## Figures and Tables

**Table 1:** Means ± SD and ranges of the erythrocyte parameters in trained subjects.

	Mean ± SD	Range
RDW (%)	13.46 ± 0.69	12.20–15.80
Ht (%)	43.88 ± 3.43	35.60–52.70
Hb (g/dL)	14.39 ± 1.16	11.70–17.50
MCV (fl)	89.00 ± 3.61	80.80–97.40
MCH (pg)	29.18 ± 1.40	26.20–33.10
MCHC (g/dL)	32.79 ± 1.18	30.40–34.80
EI 6 Pa	26.44 ± 4.30	13.57–38.03
EI 12 Pa	34.55 ± 4.54	20.55–45.57
EI 30 Pa	42.87 ± 4.68	28.30–52.64
EI 60 Pa	46.97 ± 4.53	32.82–56.10

SD = standard deviation; RDW = red blood cell distribution width; Ht = hematocrit; Hb = hemoglobin; MCV = mean cell volume; MCH = mean cell hemoglobin; MCHC = mean cell hemoglobin concentration; EI 6 = elongation index at the shear stress of 6 Pascal; EI 12 = elongation index at the shear stress of 12 Pascal; EI 30 = elongation index at the shear stress of 30 Pascal; EI 60 = elongation index at the shear stress of 60 Pascal.

**Table 2:** Means ± SD of the erythrocyte parameters in untrained and trained subjects.

	Untrained Subjects	Trained Subjects
RDW (%)	13.57 ± 0.78	13.46 ± 0.69
Ht (%)	44.15 ± 3.76	43.88 ± 3.43
Hb (g/dL)	14.47 ± 1.20	14.39 ± 1.16
MCV (fl)	86.56 ± 3.37	89.00 ± 3.61 **
MCH (pg)	28.37 ± 1.23	29.18 ± 1.40 *
MCHC (g/dL)	32.79 ± 1.15	32.79 ± 1.18
EI 6 Pa	24.08 ± 4.23	26.44 ± 4.30 *
EI 12 Pa	31.84 ± 4.51	34.55 ± 4.54 *
EI 30 Pa	39.94 ± 4.64	42.87 ± 4.68 **
EI 60 Pa	43.86 ± 4.56	46.97 ± 4.53 **

* *p* < 0.05; ** *p* < 0.01 vs. untrained subjects (Student’s *t*-test for unpaired data); SD = standard deviation; RDW = red blood cell distribution width; Ht = hematocrit; Hb = hemoglobin; MCV = mean cell volume; MCH = mean cell hemoglobin; MCHC = mean cell hemoglobin concentration; EI 6 = elongation index at the shear stress of 6 Pascal; EI 12 = elongation index at the shear stress of 12 Pascal; EI 30 = elongation index at the shear stress of 30 Pascal; EI 60 = elongation index at the shear stress of 60 Pascal.

**Table 3:** Means ± SD of the erythrocyte parameters in trained subjects subdivided according to the VO2max.

	VO_2_max < Median	VO_2_max ≥ Median
RDW (%)	13.48 ± 0.69	13.43 ± 0.70
Ht (%)	43.80 ± 3.90	43.97 ± 2.94
Hb (g/dL)	14.63 ± 1.32	14.15 ± 0.94
MCV (fl)	88.51 ± 3.90	89.48 ± 3.28
MCH (pg)	29.57 ± 1.48	28.80 ± 1.21 *
MCHC (g/dL)	33.41 ± 0.73	32.19 ± 1.23 ***
EI 6 Pa	25.21 ± 3.79	27.64 ± 4.47 *
EI 12 Pa	33.35 ± 4.20	35.72 ± 4.61 *
EI 30 Pa	41.70 ± 4.50	44.00 ± 4.63 **
EI 60 Pa	46.02 ± 4.54	47.90 ± 4.39

* *p* < 0.05; ** *p* < 0.01; *** *p* < 0.001 vs. VO2max < median (Student’s *t*-test for unpaired data); SD = standard deviation; RDW = red blood cell distribution width; Ht = hematocrit; Hb = hemoglobin; MCV = mean cell volume; MCH = mean cell hemoglobin; MCHC = mean cell hemoglobin concentration; EI 6 = elongation index at the shear stress of 6 Pascal; EI 12 = elongation index at the shear stress of 12 Pascal; EI 30 = elongation index at the shear stress of 30 Pascal; EI 60 = elongation index at the shear stress of 60 Pascal.

**Table 4:** Coefficients of correlation between age, VO_2_max, and erythrocyte parameters.

vs.	RDW	EI 6	EI 12	EI 30	EI 60
Age	−0.0361	−0.0504	−0.0367	−0.0219	−0.0156
VO_2_max	0.1052	0.2823 *	0.2493 *	0.2099	0.1676
Ht	0.0735	−0.3061 **	−0.3499 **	−0.4113 ***	−0.4342 ***
Hb	−0.0343	−0.4898 ***	−0.5263 ***	−0.5747 ***	−0.5816 ***
MCV	−0.0986	0.4945 ***	0.4847 ***	0.4554 ***	0.4439 ***
MCH	−0.2681 *	0.0794	0.0811	0.0757	0.0928
MCHC	−0.2410 *	−0.4458 ***	−0.4331 ***	−0.4081 ***	−0.3721 **

* *p* < 0.05; ** *p* < 0.01; *** *p* < 0.001 (Pearson’s test); RDW = red blood cell distribution width; Ht = hematocrit; Hb = hemoglobin; MCV = mean cell volume; MCH = mean cell hemoglobin; MCHC = mean cell hemoglobin concentration; EI 6 = elongation index at the shear stress of 6 Pascal; EI 12 = elongation index at the shear stress of 12 Pascal; EI 30 = elongation index at the shear stress of 30 Pascal; EI 60 = elongation index at the shear stress of 60 Pascal.

## Data Availability

The data presented in this study are available on request from the corresponding author. The data are not publicly available due to privacy and ethical reasons.
